# Self-inflicted Cardiac Injury with Nail Gun Without Hemodynamic Compromise: A Case Report

**DOI:** 10.7759/cureus.971

**Published:** 2017-01-10

**Authors:** Simon Ho, Bo Liu, Nicholas Feranec

**Affiliations:** 1 College of Medicine, University of Central Florida; 2 Diagnostic Radiology, Florida Hospital-Orlando; 3 Florida Hospital-Orlando

**Keywords:** nail gun, penetrating chest injury, trauma, computed tomography, cardiothoracic surgery

## Abstract

Pneumatically powered nail guns have been used in construction since 1959. Penetrating injuries to the heart with nail guns have a wide range of presentations from asymptomatic to cardiac tamponade and exsanguination. Mortality related to cardiac nail gun injuries is similar to knife injuries, estimated at 25%. Surgical exploration is the treatment of choice. We describe a case of self-inflicted nail gun injury to the chest without hemodynamic compromise in a 51-year-old man. Computed tomography (CT) imaging confirmed nail penetrating the right ventricle, with the tip adjacent to but not violating the abdominal aorta. The patient was successfully treated with thoracotomy and foreign body removal.

## Introduction

Pneumatically powered nail guns have been used in construction since 1959. Low-velocity nail guns are primarily used on wooden surfaces while their high-velocity counterparts drive nails into concrete or metal. In the medical literature, nail guns have mostly been described causing orthopedic injuries of the non-dominant arm. However, life threatening cardiac injuries have also been reported, especially self-inflicted cases [1]. Penetrating injuries to the heart with nail guns have a wide range of presentations from asymptomatic to cardiac tamponade and exsanguination. Mortality related to cardiac nail gun injuries is more similar to knife injuries than gunshot injuries, estimated at 25% [1]. Surgical exploration is the treatment of choice [2-10]. However, conservative management has been reported successful in asymptomatic cases [1]. We describe a case of self-inflicted nail gun injury to the chest without hemodynamic compromise in a 51-year-old man. Informed consent was obtained from the patient for this study.

## Case presentation

A 51-year-old white man with past medical history of depression and multidrug abuse presented to our emergency department with altered mental status and complaining of chest pain. Limited history suggested the patient was binge drinking and discharged a nail gun into his chest in a suicide attempt. However, the resulting chest pain became unbearable causing him to call a friend for transport to the emergency department. Past medical history included major depressive disorder, alcohol, tobacco and cocaine abuse, and chronic obstructive pulmonary disease (COPD). Investigations into his social history suggested his wife had died six months ago and he had been abusing alcohol, tobacco, cocaine, and marijuana heavily since. Vital signs did not suggest hemodynamic compromise: pulse was 93 bpm, blood pressure was 100/60 mmHg, and respirations were at 14 breaths/minute. The examination revealed that he was an overweight white man, weighing 75 kg and measuring 178 cm in stature. The patient was alert, although confused. A head exam showed temporal wasting and poor dental health. The pulses in the extremities were diminished but palpable, and carotid upstrokes were felt bilaterally. One puncture wound at the right sternal border between the fourth and fifth ribs was present. Additionally, the patient had decreased capillary refill and increased AP diameter. The rest of the physical exam including the cardiac and pulmonary exam were normal.

**Figure 1:**
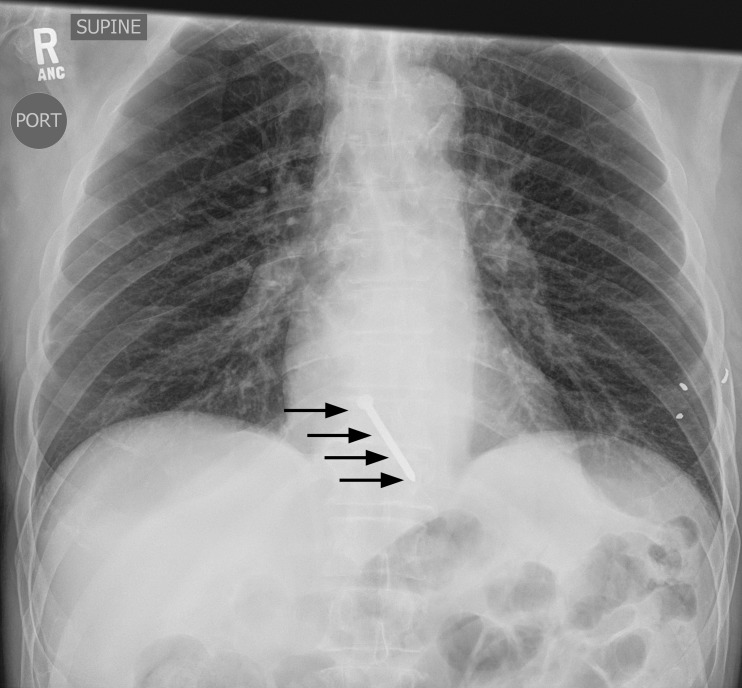
Anteroposterior Chest X-Ray Anteroposterior chest X-ray showing metallic nail overlying the heart (arrows). Also seen are buckshot overlying the left lateral chest wall and a healed left lateral rib fracture associated with a prior injury.

**Figure 2:**
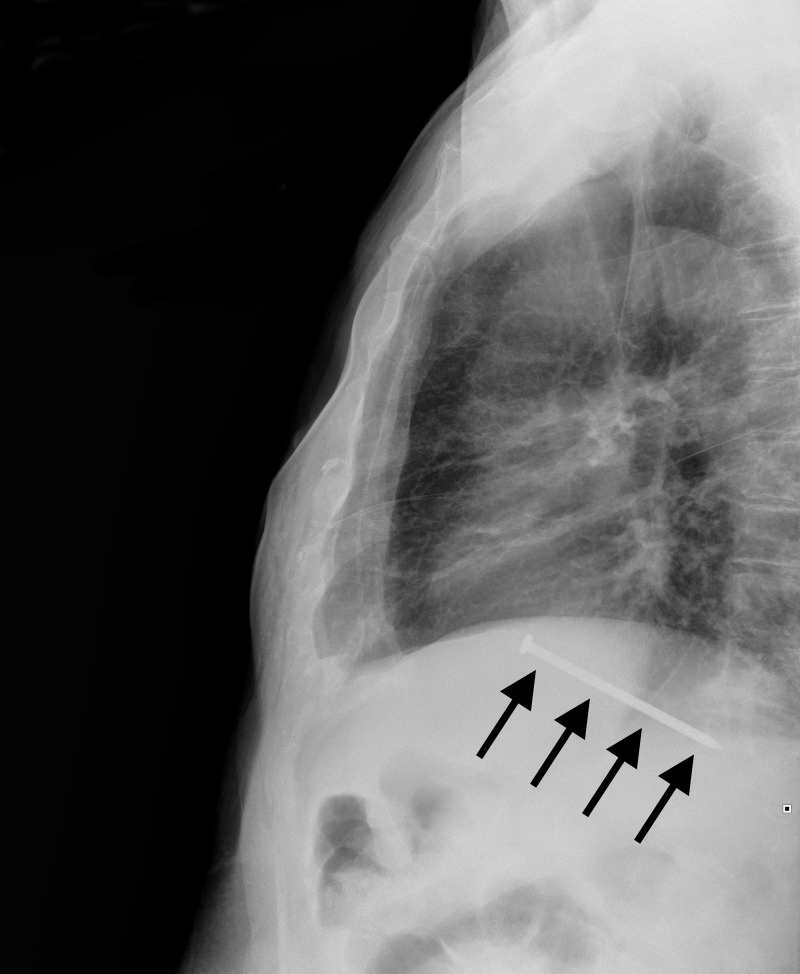
Lateral Chest X-Ray Lateral chest X-ray confirming the nail is indeed inside the thorax (arrows).

**Figure 3:**
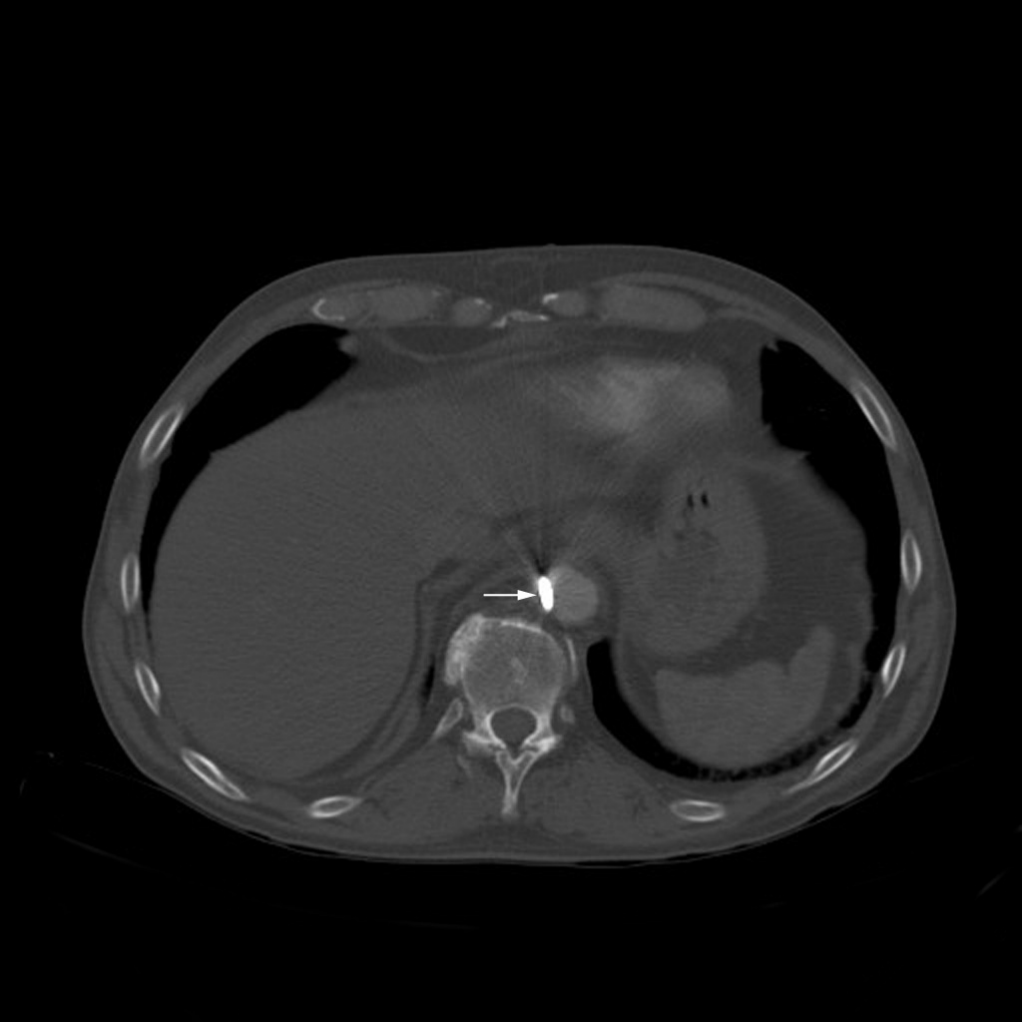
Axial contrast-enhanced computed tomography (CT) Axial contrast-enhanced computed tomography (CT) image of the abdomen showing nail tip adjacent to the abdominal aorta (arrow).

**Figure 4:**
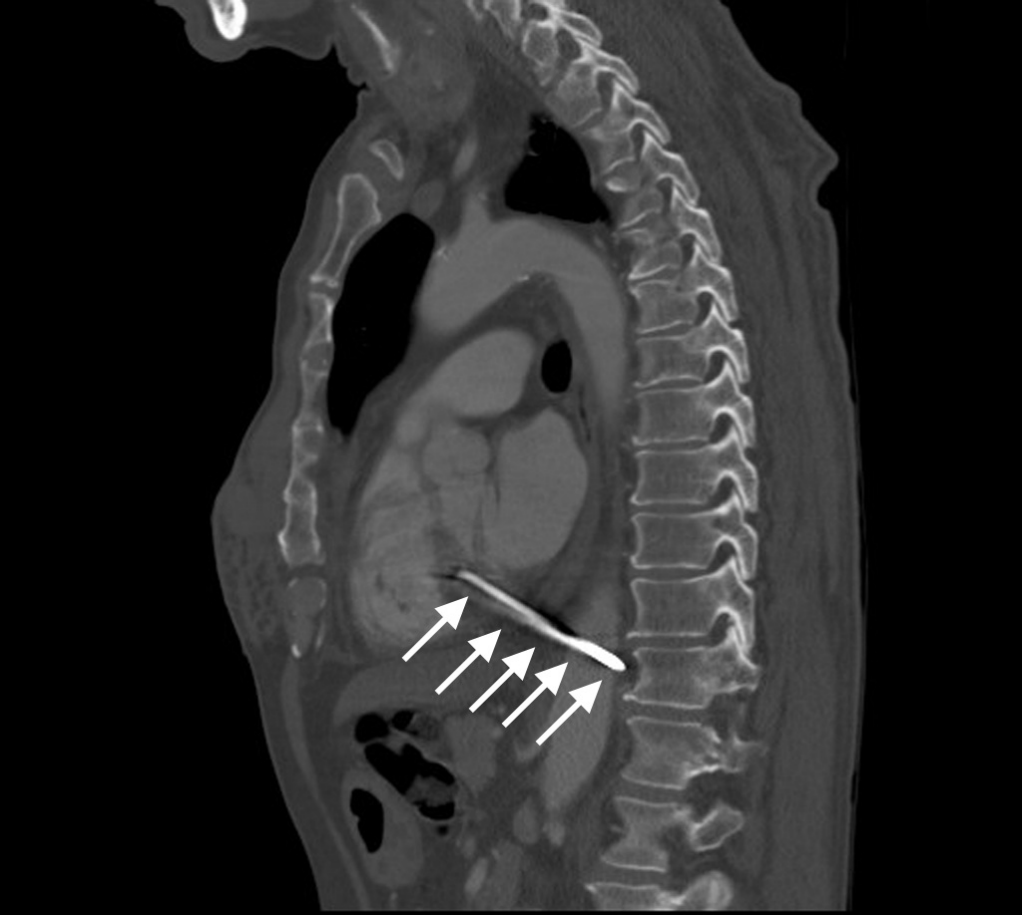
Sagittal reformatted contrast-enhanced CT Sagittal reformatted contrast-enhanced CT image showing longitudinal plane of the nail with the tip as previously described and the tail in the posterior wall of the right ventricle (arrows).

**Figure 5:**
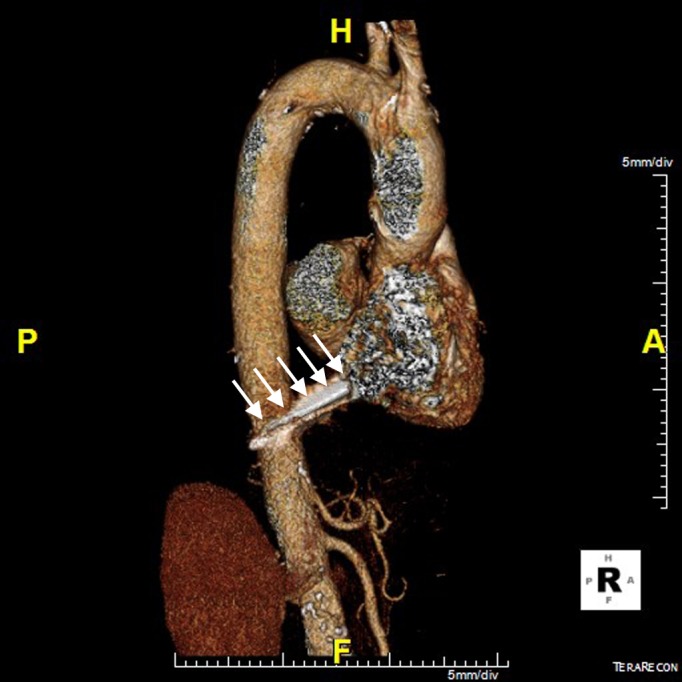
Right lateral 3D constructed contrast-enhanced CT Right lateral 3D constructed contrast-enhanced CT image showing presence of the nail puncturing the heart with the tip lying adjacent to the abdominal aorta (arrows), without evidence of rupture.

Laboratory investigations showed an elevated WBC count at 13,000/mcL and ethanol serum level at 156 mg/dL. Otherwise, his complete blood count, metabolic panel, and liver function tests were normal. Chest X-ray (Figures 1-2) and computed tomography (CT) of the chest (Figures 3-5) revealed a three-inch tapered foreign body consistent with a nail, with the tip adjacent to the abdominal aorta. The tail end of the nail was located within the right ventricular wall. Remarkably, he was not hemodynamically compromised and consented to immediate surgery for removal of the nail.

 

An intraoperative transesophageal echocardiogram confirmed the location of the nail and normal physiologic cardiac function. A median sternotomy was performed. The patient was found to have extensive pericarditis, and dissection down to the heart was required. The nail had punctured entirely through the anterior right ventricular wall, left atrium, the diaphragm, and into the abdomen, with the tail end remaining in the posterior right ventricular wall. Removal of the nail and closure of the two heart wounds and diaphragm with Prolene® Suture (Ethicon, NJ, USA) was uncomplicated. The chest was closed following the placement of two pacing wires and thoracostomy tube. Postoperative echocardiogram showed no physiologic changes from prior to removal of the foreign body. The patient did well and was extubated immediately following surgery. The patient was held for psychiatric evaluation, but was eventually discharged on postoperative day 8.

## Discussion

In the majority of cases, penetrating cardiac trauma occurs secondary to gunshot or stab wounds with reported mortality rates of 60–93% and 22–62%, respectively. Nail gun wounds are much rarer, and one case series has suggested the mortality to be close to 25%. This may be due to the smaller impact behind the projectiles relative to guns and the small frontal cross-sectional area, which focuses impact on a point. Indeed, we find several other reports similar to ours in which a patient suffering chest penetration from a nail gun, regardless of intention, had asymptomatic or delayed presentation [3, 5-6, 8, 10]. This is not to say that nail gun injuries are benign, as penetrating chest injury by nail gun has also led to catastrophic hemodynamic insufficiency [2, 4, 7, 9]. The wide range of presentations is likely due to the nail guns’ wide range of muzzle energies, lack of stabilization, and poor accuracy.

Surgery is the accepted treatment regardless of hemodynamic stability. Unstable patients should be taken immediately to the operating room. More stable patients can receive imaging such as routine chest radiograph or CT scan to help diagnose complications such as hemothorax, hemopericardium, pneumopericardium, and others [7]. CT should only be attempted on stable patients, but is particularly useful in reconstructing the track of the penetrating object and possible migration. Intraoperative transesophageal echocardiogram has been reported to be helpful in guiding surgical treatment [7, 10]. In this case, the patient was stable enough to undergo preoperative imaging, which helped guide operative therapy.

## Conclusions

Nail gun injuries to the heart, while rare, are potentially fatal and need immediate evaluation and treatment. In most cases, diagnosis can be made with history and detailed physical exam. Hemodynamically unstable patients should be operated on immediately, while imaging on stable patients can assist in the diagnosis of the penetrating agent and further complications. A CT investigation is particularly helpful in the determination of the projectile track. All patients should be considered for surgery regardless of stability. In our case, the patient was fortunate to have missed his own abdominal aorta, other great vessels, or cardiac valves, which may have led to his demise.
